# On the Absence of Chlorides in the Urine of Persons Affected with Variolous Diseases

**Published:** 1864-05

**Authors:** J. C. M. Merillat

**Affiliations:** General Hospital, Staunton, Va.


ART. II.—*On the Absence of Chlorides in the Urine of Persons affected with Variolous Diseases.* By Surg. J. C. M. MERILLAT, General Hospital, Staunton, Va.

The great importance of being able to form *an early and reliable* diagnosis in all cases resembling small-pox induces me to request the attention of your readers to the value, as a diagnostic symptom in that disease, of the presence or absence of the chlorides in the urine.

Careful observations of six cases of variola, of one of varioloid, of five cases which were supposed to be small-pox, but proved otherwise, and of three of rubeola (all that have lately occurred at this post), have led me to the following deductions, which I respectfully request those of your readers who may have more extended fields of observation to verify:

1st. That the chlorides are never present in the urine during the two first stages of small-pox.

2d. That they are absent from the beginning of the eruptive fevers—how much earlier, if at all, I have had no means of ascertaining; that, therefore, patients complaining of fever, pains in the back, etc., may be safely left in their wards, *if the quantity of chlorides in the urine be normal.*

3d. That the period of the re-appearance of the chlorides depends upon the intensity of the disease. In the distinct variety they re-appear between the eighth and fourteenth day of the eruption. Of the only two cases of confluent small-pox which I had an opportunity of observing lately, one died on the eighth day of the eruption, and in the other this day is the eleventh day of the eruption, and the chlorides have not yet returned. I am, therefore, unable to form an opinion in such cases.

4th. That the quantity of the chlorides in the urine of patients laboring under rubeola is normal during the whole progress of that disease; but it would not be safe to infer from that that every patient attacked with fever, pains in his back and head, vomiting, etc., and from whose urine the chlorides are absent, is *necessarily* laboring under the eruptive fever of small-pox, for I saw lately three patients in whom all, or nearly all, of these symptoms were present, and who recovered without any eruption. One of them says that he has never been vaccinated, and a careful examination of his arms failed to reveal any scar; neither does he know how to milk, therefore it could hardly have been a case of varioloid without eruption.

The only disease, besides small-pox and the three cases above alluded to, in which I have found the chlorides absent in the urine, is pneumonia, though I suppose further researches will swell the list.

To enable your readers to form an opinion as to the value of the observations from which the above deductions were drawn, I add the method which I have pursued in testing urine and one of the cases above mentioned:

To a small quantity of urine in a test tube, or in a clean, long and narrow phial, I add about one-sixth of its bulk of nitric acid, which I have previously ascertained not to contain any chlorohydric acid. I then add the same quantity of a strong solution of nitrate of silver, when a beautiful white curd forms, if any chlorides are contained in the urine, and more or less abundant according to their quantity. I find that only a few drops of nitric acid, as recommended by some writers, does not prevent the phosphates (often present in abnormal quantities, both in small-pox and pneumonia) from being precipitated, and they may mislead. I would prefer not adding any nitric acid than an insufficient quantity, as the phosphates can easily be distinguished from the chlorides, for a minute or two after adding the nitrate of silver, by their reddish color and floccular appearance.

Case—*Pemphigus, Variola.*—D. R. Kennedy, company "K," 14th North Carolina regiment, age 40, a farmer, was admitted October 22, 1863, with typhoid fever, on recovering from which a large abscess formed over the parotid gland. This did not heal completely till about the middle of January, 1864. He says that he was vaccinated during childhood, and that he has often seen the scar. He also says that he was re-vaccinated four times last winter unsuccessfully, though the same matter was used successfully with his children.

On January 22d, 1864, he awoke with fever and pains in his back; did not complain of headache, but of "itching" all over the body. On January 24th, an eruption of small red points on his forehead, and the same, mixed with vesicles (from quarter of an inch in diameter down), on his breast, attracted the attention of his ward surgeon, who asked for a consultation. The urine was tested and *the chlorides found to be normal.* He was ordered to be removed to a small room by himself. Jan. 25th.—*Chlorides still normal.* Eruption on the forehead rather more distinct; some of the vesicles on the breast larger (three-eighths of an inch diameter) and more opaque. On consultation with all the medical officers in the hospital, three of them expressed the belief that it was a case of pemphigus; the others believed it to be a somewhat abnormal case of small-pox. The next morning, however, on the report of some surgeons that it had assumed all the appearance of small-pox, the man was ordered to the small pox hospital. Three or four days after his admission there the eruption on his forehead disappeared entirely, whilst that on his breast went through the changes which are characteristic of pemphigus, except that the bullæ were not near as large as usual. The urine was tested every day during that time, and *invariably the chlorides were found to be normal.*

February 9th.—Awoke with severe pains in his loins, fever and vomiting, which continued the whole day. Tongue with a yellowish fur, mostly in the centre. *No chlorides in the urine.* Patient to be kept as cool as possible; no coffee or any other hot or stimulating drink. Feb. 10th.—Same as yesterday. *No chlorides in the urine.* Towards evening an eruption of minute elevated papulæ began to appear on the forehead. He was ordered a mercurial purge. Feb. 11th.—Tongue still considerably furred in the centre. Anorexia. Pains in the back continue. Eruption more distinct on the forehead. *No chlorides in the urine.* Throat sore. To use as a drink a solution of chlorate of potash, one ounce to a pint of water. Bowels have been freely moved. Feb. 12th.—Tongue still furred. Appetite returning. Throat better. Eruption very thick on the face; less so over the body. *No chlorides in the urine.* Pains in the back continue. Feb. 13th.—Has been very restless during the night. Pains in the back continue. Vesicles on the face very numerous and in some cases threaten to become confluent. Ordered the whole face to be covered with a plaster of carbonate of zinc two parts, oxide of zinc one part, rubbed into a paste with peanut oil. Throat still improving. *No chlorides in the urine.* February 14th.—Rested badly last night; frequently rubbed off parts of the plaster, which seems to have been too thin (about the consistence of thick paint). Not having any more of the carbonate of zinc, used flour instead of it. Tongue clean and moist. Appetite better. Bowels rather costive. Ordered a laxative. *No chlorides.* Feb. 15th.—Appetite moderate. *No chlorides.* Feb. 16th.—Pustules matured on the breast, and maturing on the legs, very numerous, but distinct from the knees down. Plaster covers the face completely Eyelids not swollen at all, though he says his eyes hurt him at times. Bowels regular. Appetite better. To have three ounces of wine daily. Feb. 17th.—Doing well. *Slight traces of the chlorides in the urine.* Feb. 18th.—Says he feels well to-day. No fever. The ectrotic plaster fell off in some places to-day, and was not re-applied, as the pustules under it did not look as if they would fill. Strict injunctions were left with the nurse, however, to re-apply it if he saw the least sign of their filling. *Chlorides deficient* (not absent). Phosphates abundant. Feb. 19th.—Sitting up. *The chlorides have returned,* but the urine is very turbid, and loaded with phosphates. Feb. 20th.—Continues to improve. Urine clear. *Chlorides normal.* Pustules on the face aborted; their appearance is that of large (quarter of an inch diameter) hemispheres, of natural color. Feb. 29th.—Still improving. Color of the face natural, rather bleached. No red spots like other convalescent small-pox patients. March 5th.—Discharged from the small-pox hospital, with but two "pits" about the face (doubtful whether from the small-pox), but much pitted about the body and limbs. No appearance of the pemphigus.

As somewhat connected with the above, and as it may be interesting to those of your readers who may desire to make observations on the urine in different diseases, but who, like myself, are unable in these war times to procure a microscope of high magnifying powers, I shall add that I have found one magnifying 100 linear diameters sufficient for that purpose. At least, I have repeatedly observed all the crystals (with the exception of cystine) delineated in "Bennett's Clinical Lectures" with an instrument of that power. I believe that the plates on the urine in that work ought to have been marked 75 diam. instead of "250 diam."

